# Optimisation approaches for concurrent transmitted light imaging during confocal microscopy

**DOI:** 10.1186/s13007-015-0085-3

**Published:** 2015-08-21

**Authors:** David A. Collings

**Affiliations:** Biomolecular Interaction Centre, School of Biological Sciences, University of Canterbury, Private Bag 4800, Christchurch, 8140 New Zealand

**Keywords:** Anthocyanin, Confocal microscopy, Green fluorescent protein, Köhler illumination, Plant pigments, Quantification, Real colour imaging, Transmitted light detector

## Abstract

**Background:**

The transmitted light detectors present on most modern confocal microscopes are an under-utilised tool for the live imaging of plant cells. As the light forming the image in this detector is not passed through a pinhole, out-of-focus light is not removed. It is this extended focus that allows the transmitted light image to provide cellular and organismal context for fluorescence optical sections generated confocally. More importantly, the transmitted light detector provides images that have spatial and temporal registration with the fluorescence images, unlike images taken with a separately-mounted camera.

**Results:**

Because plants often provide difficulties for taking transmitted light images, with the presence of pigments and air pockets in leaves, this study documents several approaches to improving transmitted light images beginning with ensuring that the light paths through the microscope are correctly aligned (Köhler illumination). Pigmented samples can be imaged in real colour using sequential scanning with red, green and blue lasers. The resulting transmitted light images can be optimised and merged in ImageJ to generate colour images that maintain registration with concurrent fluorescence images. For faster imaging of pigmented samples, transmitted light images can be formed with non-absorbed wavelengths. Transmitted light images of *Arabidopsis* leaves expressing GFP can be improved by concurrent illumination with green and blue light. If the blue light used for YFP excitation is blocked from the transmitted light detector with a cheap, coloured glass filters, the non-absorbed green light will form an improved transmitted light image. Changes in sample colour can be quantified by transmitted light imaging. This has been documented in red onion epidermal cells where changes in vacuolar pH triggered by the weak base methylamine result in measurable colour changes in the vacuolar anthocyanin.

**Conclusions:**

Many plant cells contain visible levels of pigment. The transmitted light detector provides a useful tool for documenting and measuring changes in these pigments while maintaining registration with confocal imaging.

## Background

Recent decades have seen a revolution in the imaging of living plant cells, with the expression of green fluorescent protein targeted to almost all locations within the plant cell [1] through different protocols including transient particle bombardment [2–4], virus-mediated infection [5, 6] and stable *Agrobacterium*-based transformations [7]. GFP expression has been visualised with advances in microscopy including the advent on confocal and two photon microscopy [8–10] and the more recent arrival of super-resolution imaging [11–13] and light sheet microscopy [14, 15]. These fluorescence techniques typically use microscopes in the epifluorescence configuration in which the excitation and emission light is delivered and collected through the same lens. In this configuration, the light transmitted through the sample can also be collected with the condenser lens and imaged with a transmitted light detector, and most confocal microscopes are now equipped with transmitted light detectors. Importantly, images detected by the transmitted light detector are recorded concurrently with confocal images meaning that they have both spatial and temporal registration with the confocal images. As a consequence of this, these transmitted light detector images can be combined with the fluorescence images into overlays in ways not possible with photographs taken with an external camera [16].

The optics of the transmitted light detector requires that the light passing through the sample to be collected by the condenser lens (rather than an objective lens), and focussed through the condenser and field irises, travelling in the reverse direction to normal, transmitted light illumination. An insertable mirror then reflects the transmitted light to the detector mounted in a conjugate plane to the lamp filament [17]. Thus, focus adjustments and modifications to the settings of these diaphragms will affect image formation. The image is non-confocal and contains out-of-focus light as it is formed by light that does not pass through a pinhole, but is does provide context for confocal fluorescence observations. Various forms of transmitted light microscopy can be achieved in association with confocal microscopy, including brightfield, polarised light, phase contrast and differential interference contrast [16]. Moreover, as these detectors do not detect the wavelength of the incident light, information about the wavelengths of light being absorbed by the sample is not recovered by the conventional transmitted light detector. For plant cells, which are often highly pigmented [18–20], this represents a loss of information.

Published confocal fluorescence microscopy images often show adjacent transmitted light images, or a superimposition of the fluorescence onto the transmitted light images. While fluorescence imaging is normally optimised, transmitted light imaging is often neglected: published images can often show excellent confocal microscopy but poor quality transmitted light images.

In this article, several approaches are documented for improving transmitted light imaging during confocal microscopy of plant cells. First, the need to optimise the transmitted light image with Köhler illumination is emphasised. Subsequently, a novel method to generate colour transmitted light images is demonstrated along with approaches to maximise image ‘resolution’ by using non-absorbed wavelengths. And finally, it is demonstrated that transmitted light image can be quantified to give meaningful and useful data.

## Results and discussion

### The importance of Köhler illumination

**Fig. 1:**
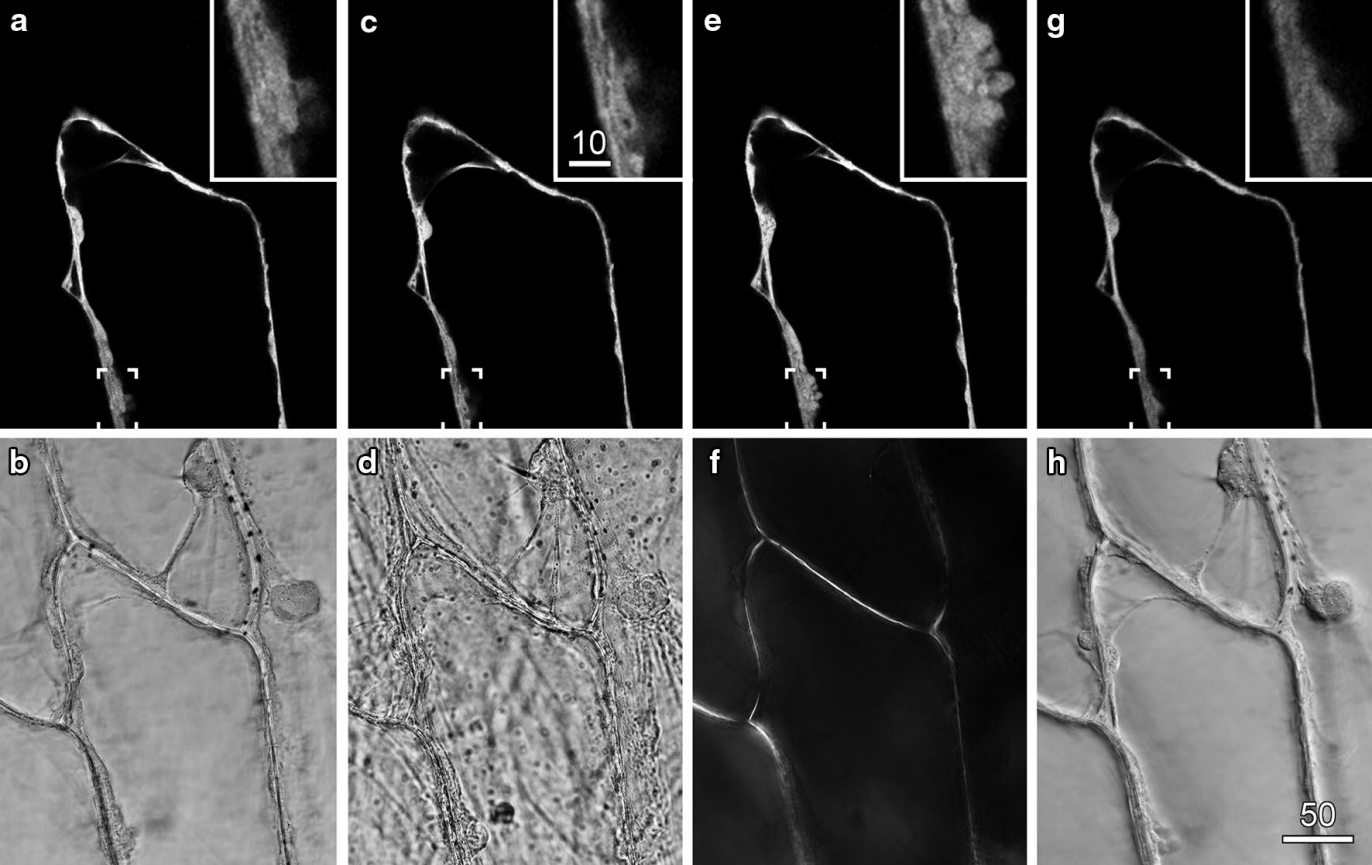
The importance of optimised illumination for transmitted light imaging. Onion epidermal cells transiently expressing a cytosolic targeted YFP were imaged by confocal fluorescence microscopy (**a**, **c**, **e**, **g**) and concurrently with various forms of transmitted light (**b, d**, **f**, **h**). **a**, **b** Optimised fluorescence and transmitted light imaging, with the transmitted light path set for Köhler illumination. **c**, **d** Non-optimal transmitted light imaging, with the condenser lens defocused and condenser diaphragm stopped down, resulted in a contrasted image with large depth-of-field. **e**, **f** Polarised light imaging revealed the location of the cell walls because of cellulose birefringence. **g**, **h** DIC imaging improved transmitted light detail, but reduced the brightness and quality of the fluorescence image as shown in the *inset* images. *Scale bars* 50 µm for main images and 10 µm for *insets*

Onion (*Allium cepa*) epidermal peels containing cells that were transiently expressing a cytosolic YFP following particle bombardment [21] were imaged by confocal fluorescence microscopy and concurrent transmitted light imaging (Fig. 1). The fluorescence image was optimised, and showed standard cytoplasmic configurations including peripheral cytoplasm around a central vacuole, and transvacuolar strands (Fig. 1a). The concurrent transmitted light image was optimised by setting the conventional transmitted light optics for Köhler illumination: this reduced the depth of field of the transmitted light image so that cellular structure within the plane of focus were more evident (Fig. 1b).

To set Köhler illumination, the following steps were adjustments were made while the sample was imaged with microscope using conventional transmitted light illumination [22–25]:

1. The field diaphragm was closed down.
2. The condenser lens was focussed so that the edges of the field diaphragm were brought into focus.
3. The condenser lens was centred.
4. The field diaphragm was opened so that the field of view was fully illuminated.
5. The condenser diaphragm was full opened.

These settings brought the microscope into Köhler illumination in which regular transmitted illumination is fully de-focused and even across the entire field-of-view. These settings are also appropriate for the transmitted light detection system associated with confocal microscopy because they will maximise collection of light, maximise the aperture of the microscope system reducing the depth-of-field, and minimise image vignetting [17]. In comparison to the optimised transmitted light image in which cytoplasmic architecture was evident (Fig. 1b), an image collected with the condenser defocused and stopped down had a poorly defined cytoplasm, showed high contrast and had a large depth-of-field in which cellular debris from the mesophyll layer, produced during the peeling process, were visible (Fig. 1d). Importantly, as the adjustments to set Köhler illumination all occurred to the light path after transmission through the sample, the concurrently-recorded fluorescence images were unaffected (except for small differences generated by cytoplasmic streaming) (Fig. 1c).

The optimisation of the light path also allows for other modes of transmitted light imaging. As lasers used for confocal microscopy are (typically) polarised [26], converting concurrent imaging to polarised light imaging only requires the addition of a polarisation filter after the sample, and does not affect the fluorescence image (Fig. 1e). Polarised light imaging provides information about the alignment of molecules within the sample, notably of cellulose within the cell wall [22, 23] (Fig. 1f), and has previously been used in conjunction with confocal imaging to look at secondary wall development in orchid roots [27, 28]. By contrast, DIC (differential interference contrast) imaging, which introduces a prism into the imaging path prior to the samples that splits the illumination light into two beams [22, 23], greatly improves the visibility of organelles and cellular contents in onion epidermis (Fig. 1g), but can cause changes in the fluorescence image. In our confocal system (but not in some other systems that have been tested), DIC resulted in a reduction in fluorescence image intensity and a loss in image sharpness at higher zoom settings (compare insets in Fig. 1g with insets in Fig. 1a, c, e). This meant that DIC images are best recorded subsequent to fluorescent images even though this results in a loss of temporal registration.

### Colour transmitted light imaging and confocal microscopy

**Fig. 2:**
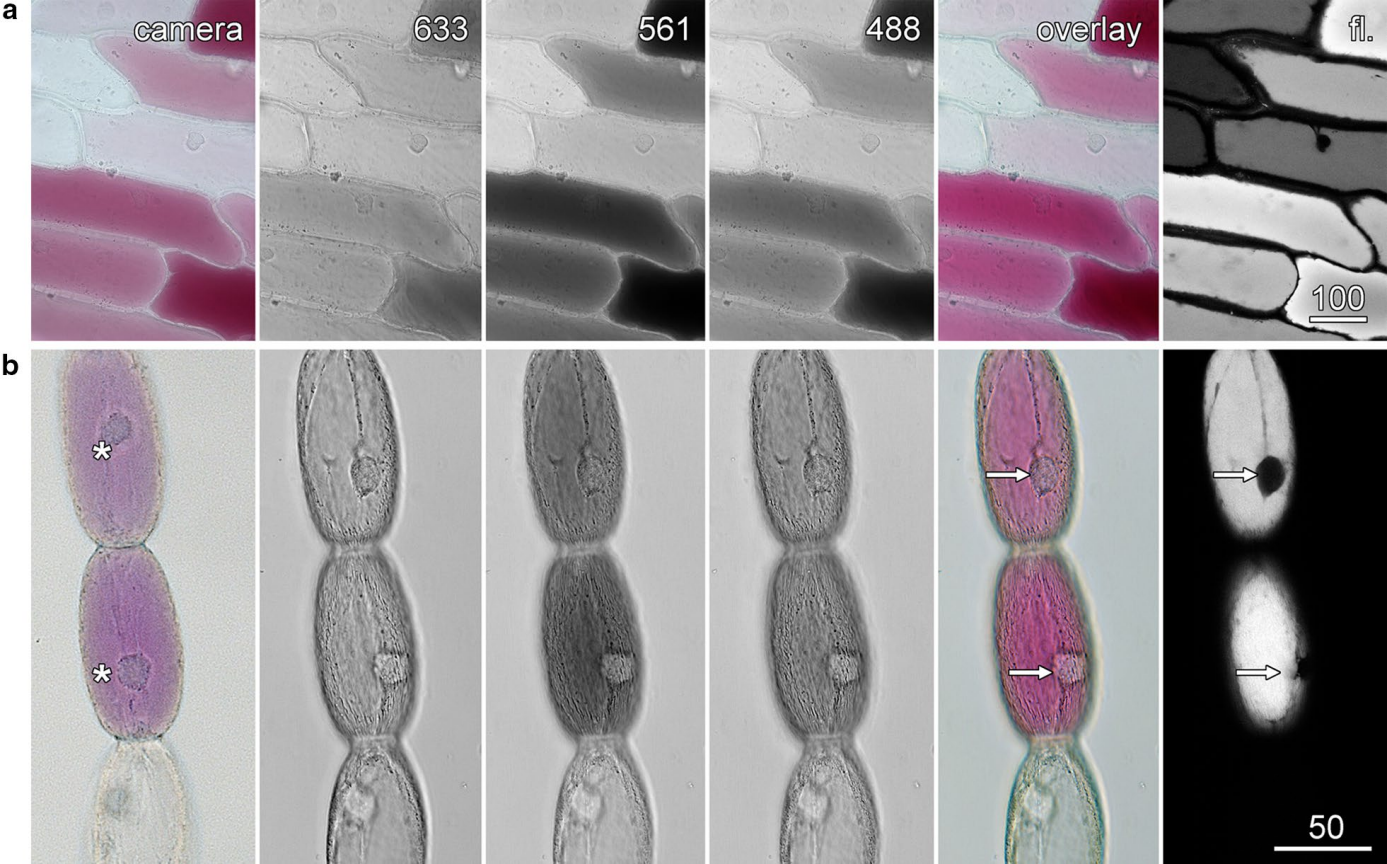
Colour images with a monochrome transmitted light detector. Transmitted light images were recorded concurrently with the 633 nm *red*, 561 nm *green* and 488 nm *blue* lasers, with these images pseudocoloured and combined into an overlay image (overlay) which matched the colour camera image (camera). Anthocyanin fluorescence images (fl.) were also collected. *Arrows* indicate nuclei in the colour transmitted light and fluorescence images, while the *asterisks* indicate the nuclei in the colour camera image that had moved between confocal and bright-field imaging. **a** Red onion epidermis. **b** *Tradescantia* stamen hair cells. *Scale bars* 100 µm (**a**) and 50 µm (**b**)

Plant cells can contain many different pigment molecules, often at concentrations that are sufficient to be visible by transmitted light microscopy. While a colour camera can record transmitted light images (Fig. 2, left panel), these images will be spatially and temporally distinct from confocal images of the same cells. Not only will the camera record a different area to the confocal image, and perhaps a different image plane if there are parfocality problems, but because it takes time to switch between the different imaging modes, the cellular contents may have moved. Therefore, the use of the confocal microscope’s transmitted light detector is beneficial as this provides image registry. Moreover, it is also possible for monochrome transmitted light detectors to record images that are, effectively, in colour through the addition of images recorded sequentially with the red, green and blue lasers [21].

Anthocyanin in the vacuole of red onion epidermal and *Tradescantia* stamen hair cells was imaged by a conventional colour camera (Fig. 2; left column), and by fluorescence (right column) with the confocal microscope. Separate, non-confocal images were also recorded with the confocal system’s transmitted light detector using the 633 nm red laser, the 561 nm green laser and the 488 nm blue laser.

In the Leica SP5 confocal system, there are several options for separately and sequentially scanning images with multiple lasers. Sequential images can either be collected in frame-by-frame mode, in which the imaging laser scans the entire image before being switched to the next imaging laser, or in line-by-line mode in which single lines of the image are collected sequentially for each laser before moving on to the next line of the image. For living plant cells, which often show dynamic cytoplasmic streaming, line-by-line imaging is preferable for generating the red, green and blue transmitted light images as it improves the alignment of the three images: in frame-by-frame imaging, streaming can result in movement between the individual images. The use of line-by-line scanning, however, imposes a significant limitation on the transmitted light detector, as the detector’s offset and gain settings cannot be modified for the different lasers. Instead, it is necessary to modulate laser strength to generate red, green and blue images of roughly even intensities. For our Leica SP5 confocal, laser powers of about 100, 30 and 3 % provide a balanced composite image when summed from the 633, 561 and 488 nm lasers, respectively, although these values will vary between confocal microscopes because of differences in the type and age of the lasers. In such cases, the easiest way to balance the lasers is to optimise the transmitted light image for the weakest laser (typically the 633 nm red laser). The gain on the transmitted light detector is then set so that the background just reaches saturation, identifiable using an appropriate look-up-table. The power of the remaining two lasers can then be adjusted so that they too give images that just reach saturation. Lowering the transmitted light detector gain should then give red, green and blue transmitted light images of approximately equal intensities in which variations between the images correspond to the absorbance of light by the sample.

**Fig. 3:**
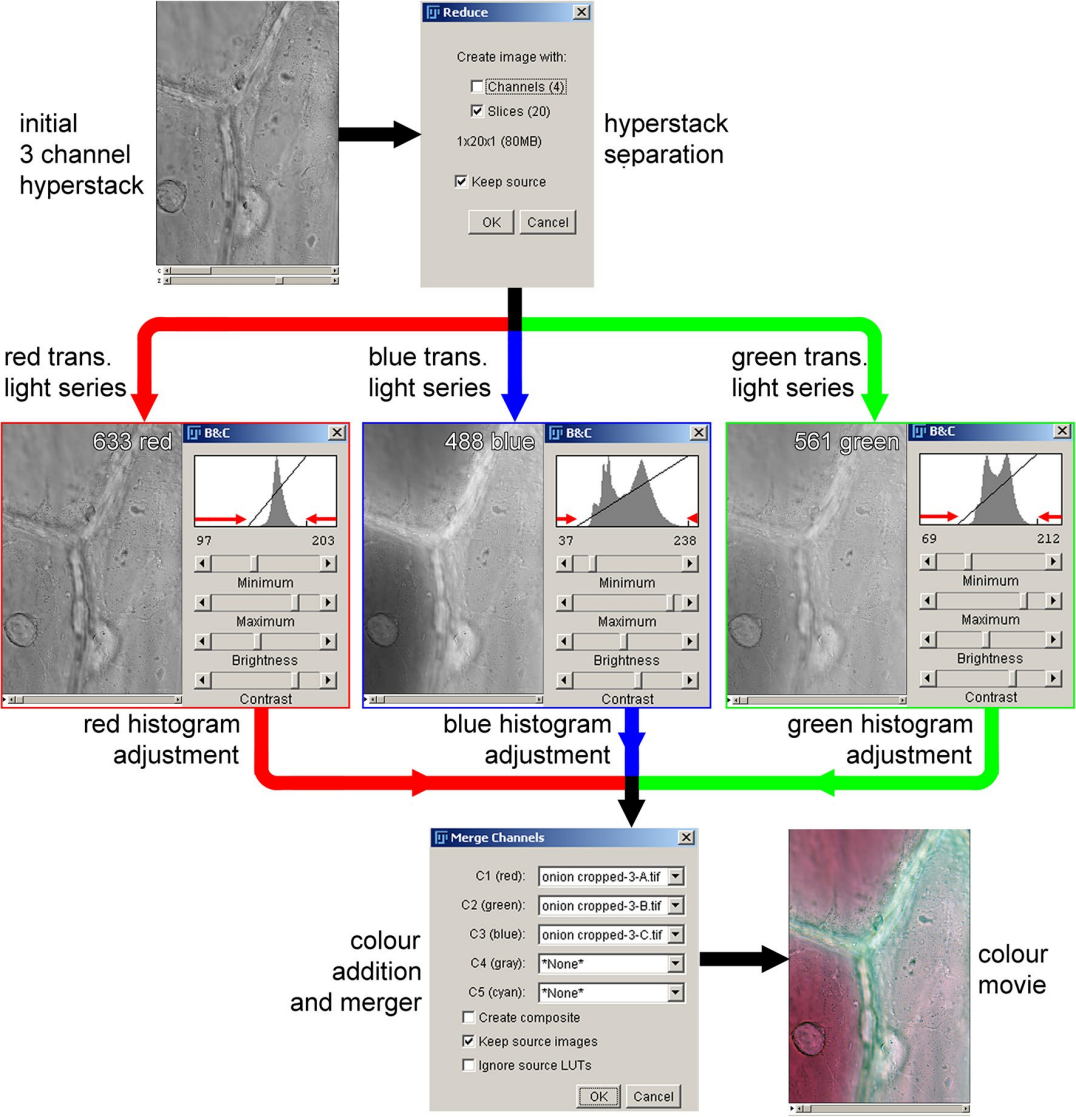
Generating colour transmitted light images in ImageJ. Balanced colour images can be generated from individual images or image stacks using a simple procedure in ImageJ, even if the images recorded for the 3 different wavelengths were of different intensities. A confocal hyperstack showing onion epidermal cells (3 transmitted light channels imaged over time) was opened in ImageJ. These were separated into image stacks representing the 633 nm *red*, 561 nm *green* and 488 nm *blue* transmitted light series using the command ‘reduce dimensionality’ (in the ‘hyperstacks’ option in the ‘image’ menu). For each of the stacks, the histogram was adjusted using the ‘brightness/contrast’ command (in the ‘adjust’ option in the ‘image’ menu). This involved adjusting the minimum and maximum values on the histogram to match the edges of the histogram, as indicated by the *red arrows*. A combined colour image was then generated using the ‘merge channels’ command (in the ‘color’ option in the ‘image’ menu). *Scale bar* 50 µm

While the separate red, green and blue transmitted light images can be combined into a colour image in numerous different programmes, including the Leica confocal software, processing the images in ImageJ allows alteration to the colour balance of images while retaining the ability to work with image stacks. Confocal images can be opened in the FIJI installation of ImageJ (http://fiji.sc/Downloads) as a hyperstack (a multichannel set of image stacks), and these can be separated into image stacks representing the 633 nm red, 561 nm green and 488 nm blue transmitted light images, along with any fluorescence images, using the ‘reduce dimensionality’ command in the ‘hyperstacks’ option of the ‘image’ menu. The transmitted light images can then be recombined into a colour image using the ‘merge channels’ command found under the ‘color’ option in the ‘image’ menu (Fig. 3).

In the onion epidermal and *Tradescantia* stamen hair cells, this type of imaging generated 3 colour-specific transmitted light images in which the anthocyanic cells strongly absorbed both blue and green light, but did not absorb red light (Fig. 2). When merged to produce colour images, these matched those taken with the colour camera (Fig. 2, overlay). Importantly, these images were in register in both time and space with confocal fluorescence images (Fig. 2, fl.). This was best demonstrated by the nuclei in the *Tradescantia* cells that were in the same location in the colour transmitted light and fluorescence images, but which were in different locations in the colour camera image because of the highly active cytoplasmic streaming present in these cells (arrows).

ImageJ can also be used to correct images collected when the lasers and the three resulting transmitted light images were not well balanced. Imaging under these conditions results in a coloured background and incorrect representations of coloured objects within the sample. This correction process, shown in Fig. 3, involves normalising the image histogram, the graphic representation that shows the distribution of the number of pixels with different intensities within the image. This process can either be run on merged colours images or on the individual images prior to merger. To colour balance before merging the images requires adjusting the image histogram with the ‘brightness and contrast’ command in the ‘adjust’ option in the ‘image’ menu. The sliders for the minimum and maximum value need to be adjusted to match the visible edges of the histogram, as shown by the red arrows in Fig. 3. To colour balance an already merged image, the ‘color balance’ command in the ‘adjust’ option in the ‘image’ menu is required. The image histogram is then adjusted for each colour (red, green and blue). It is, however, necessary to ‘apply’ any adjustments made in one colour before moving to the next colour. An advantage of working with already merged images is that this allows a comparison of the overall colours to be observed while the changes are being made. The adjustments to the image histogram introduced by ImageJ are linear modifications, and are usually all that are required to generate a satisfactory colour recombination. More complex and precise methods for image histogram modification and colour balancing, often involving non-linear modification to the histogram, are discussed in reviews of image processing (see, for example pages 274 onwards in [29]).

Several further suggestions on colour transmitted light imaging with confocal microscopy can be made:

1. Image collection is slow, especially if multiple fluorescence channels are also recorded using different laser powers, so image optimisation is best at low resolution (e.g. 256 × 256 pixels) with only final images and movies recorded at higher resolutions.
2. Colour imaging in the transmitted light mode is also useful for fixed and stained samples [30] where the maintenance of spatial registration remains important even if temporal registration is not longer problematic. In fixed samples, the frame-by-frame option for sequential scanning may provide slightly faster imaging, along with the added advantage that gain values can be individually optimised for the red, green and blue lasers.

This approach to colour transmitted light imaging in confocal microscopy, via use of sequential scanning of red, green and blue lasers, does not appear to have been previously used although colour reflected light confocal microscopy, based on the addition of red, green and blue lasers has previously been described [31]. This additive approach to colour generation appears to be different to a previous approach to real-colour transmitted light imaging: some versions of the Bio-Rad MRC1000 confocal system had a colour transmitted light detector that worked by passing light through red, green and blue filters in front of the detector [16, 32].

### Optimising the wavelength for transmitted light imaging

In many cases, it is neither necessary nor desirable to collect colour transmitted light images. However, in imaging pigmented or thicker samples where light transmission may be poor, consideration should be given to the correct wavelength with which to record the transmitted light images.

**Fig. 4:**
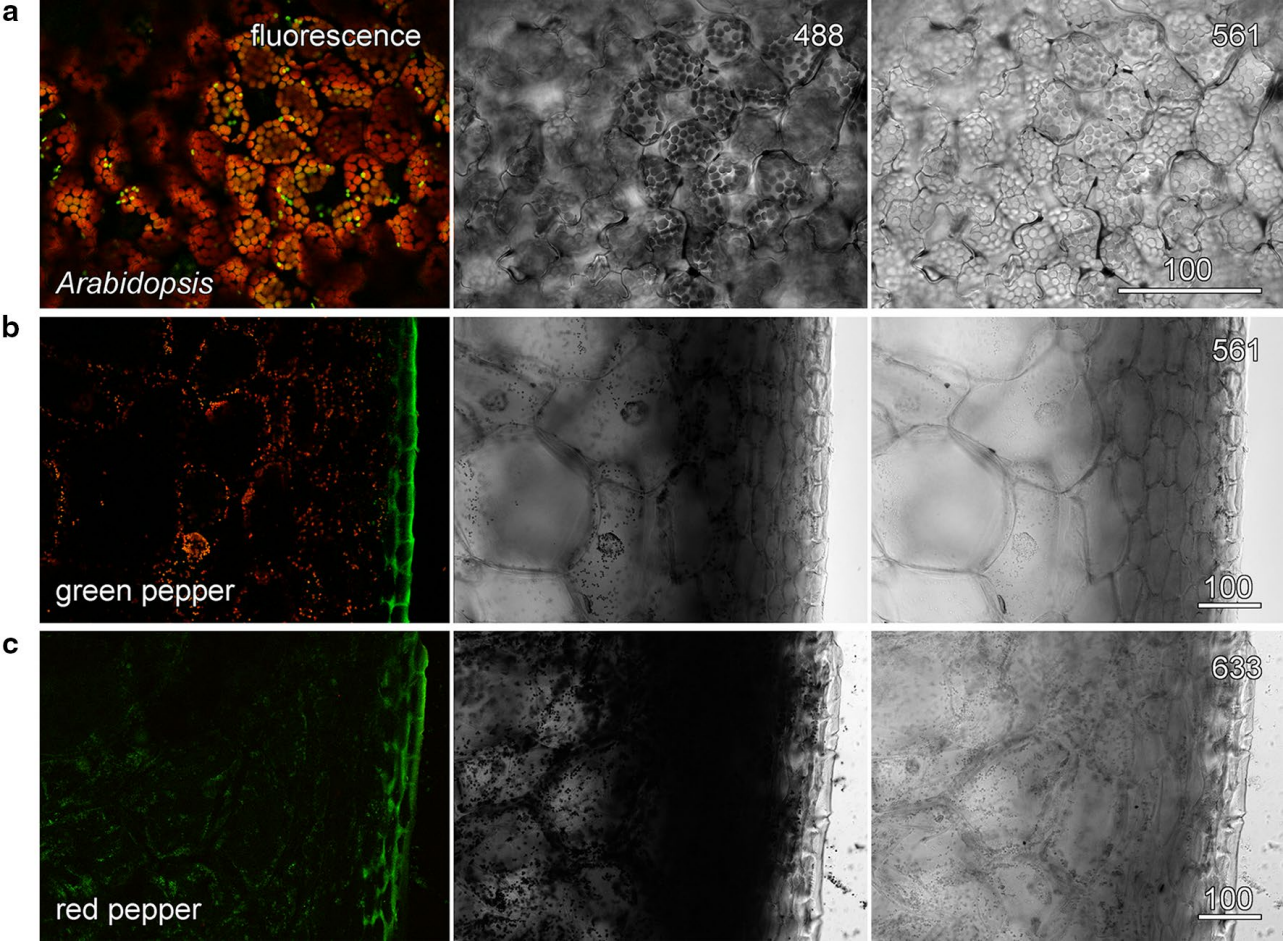
Concurrent transmitted light images were improved by non-absorbed lasers. In thicker and more absorbing samples, transmitted light images were optimised using non-absorbing wavelengths. **a** *Arabidopsis* leaf expressing plastid-targeted GFP. **b** *Green* pepper pericarp section. **c** *Red* pepper pericarp section. Fluorescence images show chlorophyll (*red*) and either YFP (**a**) or a combination of carotenoid and cuticle autofluorescence (**b**, **c**) (*green*). Transmitted light images were recorded with 488 nm blue light which was absorbed strongly by chlorophyll and lycopene (central column) or with 561 nm *green light* (**a**, **b**) or 633 nm *red light* (**c**) which were not strongly absorbed. A colour filter was used to remove any transmitted *blue light*. *Scale bars* 100 µm

Confocal imaging of chlorophyll autofluorescence (red in overlay) and YFP targeted to plastids (green in overlay) in the epidermis and palisade mesophyll of whole *Arabidopsis thaliana* leaves used 488 nm blue excitation and resulted in poor transmitted light images because of the very strong absorbance by chlorophyll (Fig. 4a). When the transmitted light images was recorded concurrently with the 561 nm green laser, a more even, information-rich image was possible because the green laser was not absorbed strongly by the leaf. Although the green light imaging might be done in sequential scan mode, or achieved with high laser intensities to over-power the blue laser with the possibility of laser-induced tissue damage, the easiest method is to filter out the remaining blue light after it has passed through the sample. Relative transmission rates for camera filters of different colours, often available with defined spectral characteristics and for sale second-hand in camera stores, were measured using the transmitted light detector (Table 1). An orange filter (details) that transmitted green light but blocked blue light greatly improved the transmitted light image of the leaf (Fig. 4a). Importantly, the only effect on the fluorescence imaging of including the green laser in the system was an increase in chlorophyll autofluorescence. Similar effects can be generated with coloured filters used for stage lighting and for which published spectra are usually available.

**Table 1:** Transmission rates for colour photographic filters

Laser wavelength (nm)	Red (Hoya 25A)	Orange (Marumi MC-YA2)	Yellow (Hoya K2)	Green (Hoya G)
405	0.1	0.1	0.1	0.1
458	0.1	0.2	0.2	2.3
476	0.2	0.2	0.6	10.9 ± 0.3
488	0.1	0.1	24.5 ± 0.8	19.7 ± 0.6
496	0.1	0.1	58.2 ± 1.5	27.5 ± 1.0
514	0.1	0.5	84.5 ± 2.2	43.9 ± 1.8
561	0.1	33.0 ± 1.1	90.2 ± 1.6	38.5 ± 1.2
633	93.4 ± 2.8	93.9 ± 2.8	89.4 ± 1.6	3.3

Transmission rates were calculated as the percentage transmission through filters compared to control values without filters. Values are means of four replicates, with SEM values shown for transmission rates above 5 %

The use of coloured filters was also tested using hand sections through the pericarp (outer, firm layer) of green and red peppers (*Capsicum annum*). In both cases, the 488 nm blue laser was strongly absorbed by chlorophyll or lycopene in the plastids. Transmitted light imaging of green pepper required the 561 nm green laser and the orange filter (Fig. 4b) whereas the 633 nm red laser and a red filter were used for good transmitted light imaging of the red pepper (Fig. 4c). The 633 nm laser, having a lower energy, is less likely to cause tissue damage. Another instance in which coloured filters have been useful for transmitted light imaging is in the elimination of laser noise, sometimes present with older lasers. The use of the stable 633 nm red laser, and either a red or orange filter, can overcome such problems.

### Improving light transmission in plant samples

The use of an appropriate wavelength is not the only way that transmitted light images can be improved in thick plant samples. The irregular air spaces within plant leaves present around mesophyll cells diffract light as it passes through the leaf, and reduce the quality of transmitted light images by eliminating air spaces, either with water [33, 34] or perfluorcarbon oils [35, 36], refraction is reduced and transmission increased.

**Fig. 5:**
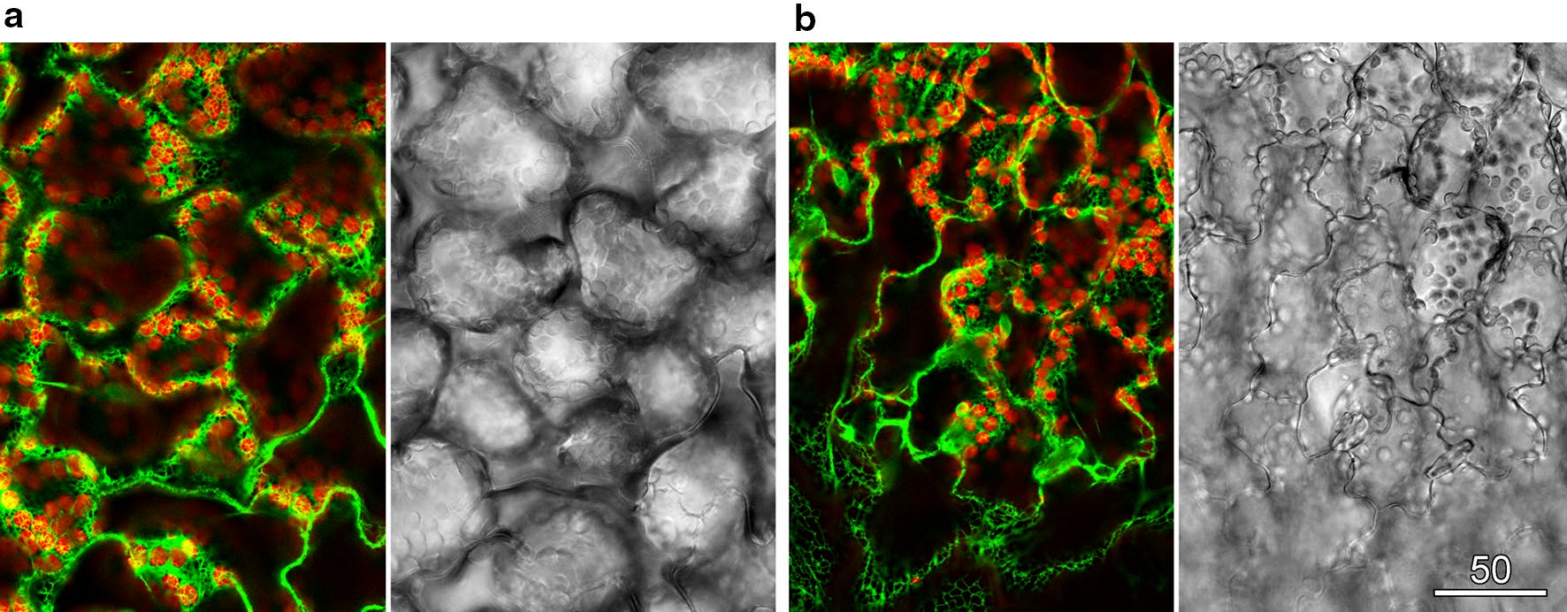
Water infiltration through de-gassing improved transmitted light imaging of leaves. Images show the epidermis and palisade mesophyll of a young *Arabidopsis* leaf, and are fluorescence with an overlay of YFP-HDEL (*green*) and chlorophyll autofluorescence (*red*) and 561 nm *green* transmitted light images. **a** Control. **b** Leaf de-gassed (2 × 5 min)—the fluorescence images were unaffected by the de-gassing treatment which removed air pockets behind the image plane. *Scale bar* 50 µm

The epidermis and palisade mesophyll of young *Arabidopsis* leaves expressing ER-targeted YFP were imaged. was present in its reticulate cortical arrays, subcortical and transvacuolar strands and in the nuclear envelope, but transmitted light imaging with 561 nm green light was poor because of the presence of air pockets within the leaf (Fig. 5a).Vacuum infiltration of water (2 × 5 min) drew air out of the leaf and replaced it with water, and greatly improved the imaging (Fig. 5b). This process does not modify the fluorescence image of the epidermis and upper mesophyll because the air pockets lie behind these planes, although fluorescence imaging further into the leaf would be greatly improved [35]. Degassing also improved imaging of simple systems such as onion epidermal peels. When small gas bubbles caught on the surface cuticle layer of the epidermis where removed, transmitted light imaging improved.

### Quantification of transmitted light images can reveal further information

Colour transmitted light imaging on the confocal microscope was refined for imaging red onion epidermal cells [21] where multiple-wavelength transmitted light images have quantified anthocyanin development and function [37, 38]. In these studies, vacuolar anthocyanin was measured as the ratio of the transmitted light image intensities recorded with the non-absorbed 633 nm red laser divided by the absorbed 561 nm green laser. However, all the laser wavelengths contain information about the anthocyanin content within the vacuole and, moreover, information about the vacuole itself.

**Fig. 6:**
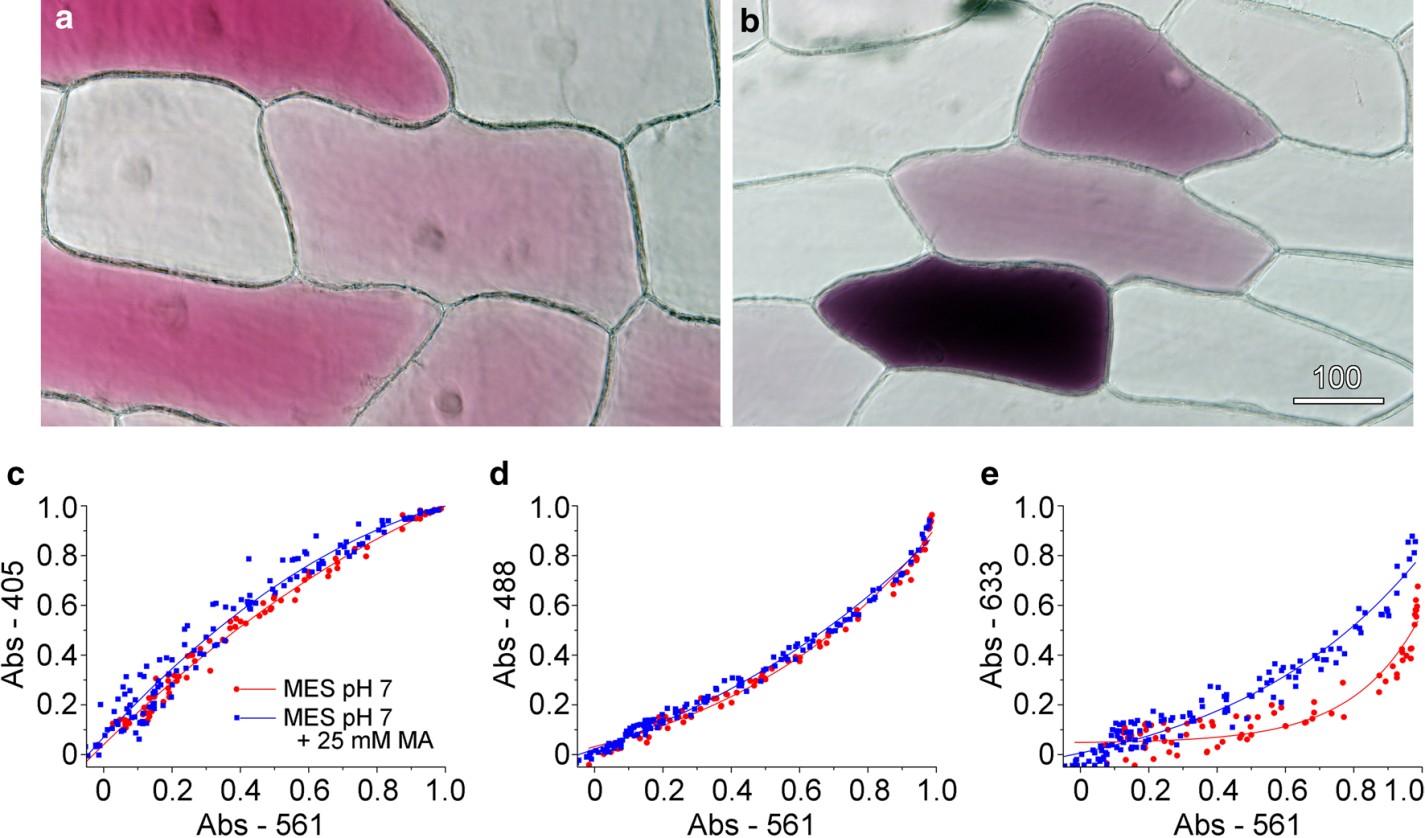
Transmitted light imaging quantified changes in vacuolar pH. Red onion epidermal cells were incubated on 40 mM MES at pH 7, or on this buffer containing 25 mM methylamine (MA), and were imaged with 6 different wavelengths (405, 456, 488, 514, 561 and 633 nm). **a** Colour transmitted light imaging after incubation of buffer alone. **b** Colour transmitted light imaging after treatment for 10 min with 25 mM methylamine. **c**–**e** For each of the wavelengths and for each cell, a vacuolar absorbance value was calculated. This was defined as 1 minus the background-corrected transmission of the laser, relative to a cell-free reading. Absorbance values for cells at 405 nm (**c**), 488 nm (**d**) and 633 nm (**e**) correlated to absorbance measured at 561 nm. Each *dot* represents a different cell, and data were fitted with quadratic (**c**, **d**) and exponential equations (**e**). Absorbance of *red light* (**e**) was much stronger in the MA-treated cells, consistent with the change in colour

As anthocyanins show pH-dependent changes in colour, typically shifting from red to blue as pH increases [19, 20], pH responses in the vacuole were triggered and measured. The pH of the vacuole can be modulated by the addition of membrane-permeant exogenous weak bases such as methylamine. Being uncharged, methylamine crosses through the plasma membrane and tonoplast but is acidified on entering the vacuole. The accumulation of protonated methylamine within the vacuole reduces the H^+^ concentration in the vacuole transiently making the vacuolar pH less acidic, although the cell can recover an acidic vacuole over time [39, 40]. Transmitted light images were recorded at 6 different wavelengths (in 2 groups of 3) for red onion inner epidermal cells that had patches of anthocyanic cells. The images at 488, 561 and 633 nm could be used to generate colour transmitted light images that demonstrated the variable nature of the red patterning (Fig. 6a). Absorption values were calculated for all 6 wavelengths for the vacuole of each cell. When absorbance values that were measured for control cells for 405 nm violet (Fig. 6c), 488 nm blue (Fig. 6d) and 633 nm red light (Fig. 6e) were plotted against the absorbance values for 561 nm green light, tight correlations were always observed. The other wavelengths measured (456 and 514 nm) showed similar correlations (data not shown). As would be expected for the presence of a single main pigment in the vacuole, these correlations were linear except at very high absorbance when linearity was lost.

When red onion was floated on 25 mM methylamine at pH 7, the epidermal peels rapidly changed from red to dark blue or black. Colour transmitted light imaging confirmed the colour change with cells showing a darker and bluer appearance. Quantification of absorbance ratios showed no or limited changes at 405 and 488 nm, respectively (Fig. 6c, d) but significant increases in absorption at 633 nm (Fig. 6e) consistent with cells being less red. Cells remained alive during these treatments and cytoplasmic streaming continued.

## Conclusions

This paper has described ways in which transmitted light imaging can be optimised (Köhler illumination) and extra information gathered (use of non-absorbed wavelengths for imaging, generation of coloured images). Applicability of absorption measurements has also been demonstrated, initially at a small range of wavelengths. The development of tuneable, visible wavelength lasers (white light lasers) ([41], http://www.leica-microsystems.com/science-lab/white-light-laser) covering from 470 to 650 nm opens up prospects of cellular level, micro-spectroscopy, a process that might prove to be extremely useful for coloured plant specimens.

## Methods

### Microscopy

A Leica SP5 confocal microscope system operating on an DMI6000 inverted microscope and equipped with 20× NA 0.7 and 63× NA 1.3 glycerol immersion lenses (Leica, Wetzlar, Germany) was used for this study. The confocal was equipped with 4 different lasers that could provide excitation at 405 nm (violet), 458, 488 and 514 nm (blue), 561 nm (green) and 633 nm (red), and had a transmitted light detector. Living plant tissue was mounted in water, except as noted, and under a coverslip in uncoated FluoroDishes (35 mm diameter, WPI, Sarasota, FL USA) or on microscope slides. Onion epidermis was mounted with it cuticle towards the lens and *Arabidopsis* leaves mounted with their upper, adaxial surface towards the lens. Fluorescence images for GFP, YFP and carotenoids were collected using 488 nm excitation, with emission from 500 to 550 nm, while red fluorescence from anthocyanin and chlorophyll was excited at 561 and 488 nm, with emission set to 570–620 and 650–700 nm respectively. Images were recorded at high resolution (either 1024 or 2048 pixels square) using 4-fold or higher line averaging.

For excitation with multiple wavelengths and the generation of wavelength-specific transmitted light images, sequential imaging was used in line-by-line mode. Each line of the image was recorded with one laser line, and then subsequently re-recorded with one or more different wavelengths. Alternatively, multiple excitation wavelengths could be used with one or more of these wavelengths filtered from the light path after it had passed through the sample but before it reached the transmitted light detector through the addition of coloured glass filters. These coloured glass filters were purchased second-hand from local camera stores.

### Image modifications

All image modifications used standard brightness, contrast and gamma settings in Photoshop (version CS4, Adobe Systems, San Jose, CA, USA) and ImageJ (FIJI installation of version 1.47v, National Institute of Health, Bethesda, MD, USA). Image intensities were quantified from image stacks in ImageJ by selecting a region (usually the vacuole) with quantification using the ‘Plot Z axis profile’ function.

### Plant material

Red onions and peppers were bought from local supermarkets, with transient gene expression of cytosolic YFP in onion epidermal cells conducted as previously described [21]. Epidermal peels were prepared from 10 to 15 mm square pieces of inner epidermis by carefully lifting the epidermis away from the underlying mesophyll with a pair of tweezers. For pH modification experiments, epidermal peels were floated on a solution of 25 mM methylamine in 40 mM MES at pH 7.0 (5–10 min) and then mounted in this solution. Controls were pretreated and mounted with the MES buffer only.

Flowers from *Tradescantia* sp. were collected from plants grown in the University greenhouses. *Arabidopsis thaliana* lines expressing YFP targeted to the ER and plastids was obtained from the Arabidopsis Stock Center (lines CS16252 and CS16267 [42]). Seed was surface sterilised and sown on 1.2 % agar plates containing 3 % sucrose and Hoagland’s solution, and plants were grown at 21 °C under 24 h light (100 µE m^−2^). Expanding leaves were used when the plants were 2 weeks old. To de-gas the young leaves, entire leaves were placed in 1.5 ml Eppendorf tubes which had a hole punched in the lid and which were completely filled with water. Closing the lid forced the leaf sample into the liquid. Water was forced into the leaves by pulling a vacuum (2 × 5 min).

Experiments with genetically modified plants were carried out in accordance with New Zealand regulations, and under permits GMC08010 and GMD08056.
